# Training in management of arrhythmias for medical residents: a case-based learning strategy

**DOI:** 10.5116/ijme.57c2.a738

**Published:** 2016-10-05

**Authors:** Daniel Rodriguez Muñoz, Gonzalo Alonso Salinas, Eduardo Franco Diez, Javier Moreno, Roberto Matía Francés, Antonio Hernández-Madrid, José Zamorano

**Affiliations:** 1 Cardiology Department, University Hospital Ramón y Cajal, Madrid, Spain; 2 Medical School, University of Alcalá de Henares, Madrid, Spain

**Keywords:** Residency, case-based learning, training strategies, arrhythmias.

## Introduction

Continuous medical training (CMT) has proved to be an effective method to improve and sustain professional medical competence.^1^^,^^2^ Consequently, several studies have assessed the effectiveness and the impact of different strategies and educational methodologies applied to CMT. However, the available evidence in this context is relatively scarce, limiting the possibility to draw definitive conclusions. Some studies have suggested that, among the different approaches, those that generate a higher impact in learners and effectively change their clinical practice are those based on interactive methodologies, mainly those using simulation and case-based learning as pivotal learning elements.^1^^,^^3^^,^^4^ Additionally, the audience in postgraduate CMT seems to show a more positive attitude towards these interactive programs that engage students in their learning process assigning them an active role.^4^^-^^6^ Despite this, traditional training strategies based on presentations and lectures are still predominant.^3^ The use of cases or problems as the basis of learning strategies is more common in pre-graduate training.^7^ However, its use in post-graduate training and long-life-learning is rare in spite of evidence suggesting stronger learning outcomes.^3^^,^^7^^-^^10^

### Previous curriculum: room for improvement

A four-day course is organised yearly in our centre to train medical residents in the diagnosis and management of suspected acute cardiac conditions related to arrhythmias in the emergency department. The teaching methodology of the course was mainly based on lectures with the occasional use of clinical cases to foster interaction among learners and between them and teachers.

The evaluations we received from the participating residents after each course were positive. However, there was a generalized perception among those implicated in designing and carrying out the course, as well as among cardiology staff more in contact with the emergency department, that the effect of these training courses in residents’ performance was important but short-lived, with an early regression to lower levels of competence and quality of care shortly after its conclusion.

### Changing the curriculum

We aimed to improve the residents’ clinical performance in a way that had a more durable effect in their practice. We thought that this would be better achieved through an active process of reflection and search for answers by the learners instead of passively listening to presentations and seminars. Consequently, we worked on the idea of changing the curriculum into a case-based training methodology.

Before changing the whole structure of the course, we wondered which items would benefit more from an interactive, case-based strategy. Consequently, we designed an initial experience in which we divided medical residents to participate in the case-based learning strategy or conventional, seminar-based course, for the part of the course covering atrial fibrillation, syncope, and cardiac stimulation devices.

This way, we tested the new curriculum, based on the use of clinical cases or open issues specifically designed to stimulate the residents’ reflection and to lead them to formulating relevant questions. Time was allocated for reading of the problems, reflecting, and writing questions related to them; “protected time” dedicated to literature review to answer the generated questions; and, finally, a joint group discussion, conducted by two trainers, based on the questions and answers generated in the previous two phases.

Fifty days after the end of the course, we asked the residents to take a test to try to assess not the immediate effect of the course on the residents’ knowledge, but its potential to improve retention of knowledge and clinical reasoning abilities long after its conclusion. The proportion of residents who took the voluntary test was much larger in the group participating in a case-based strategy, and they obtained better results in questions related to atrial fibrillation and syncope but worse in those related to cardiac stimulation devices. We also asked the participants to answer a course evaluation questionnaire right after its end. Results did not differ between the two groups in their global rating of the course or in any of the specific items evaluated.

### Lessons learned

This experience showed us that it is feasible to organise and carry out this type of course for residents following a case-based strategy. The data from the test and the evaluation questionnaire taught us that residents became more involved and proactive in their learning process and, consequently, more eager to test their competence, when following a case-based strategy. This experience encouraged us to design large parts of our training courses following a case-based strategy. We believe that this strategy may produce better results in knowledge retention and ability to resolve clinical problems in areas more oriented to clinical reasoning and decision-making.

We also observed that this training methodology is more complicated and may be less adequate when the discussion of as many different cases and scenarios is more important than a deeper reflection on fewer problems. This may be the case in issues related to cardiac stimulation devices, where we encounter a vast range of different cases and visual support is key in providing a reasonable number of examples.

## Conclusions

The application of a case-based learning strategy is feasible and effective for training of medical residents in management of arrhythmias, but may be less useful in aspects that require stronger visual support or that may benefit from the visualization and discussion of several cases rather than a deeper reflection on fewer clinical scenarios. Medical residents value this learning strategy similarly to seminar-based programmes, in spite of its longer duration. Additionally, case-based training appears to increase the residents’ implication in their own their learning process.

### Conflict of Interest

The authors declare that they have no conflict of interest.
